# A multisource fusion framework driven by user-defined knowledge for egocentric activity recognition

**DOI:** 10.1186/s13634-019-0612-x

**Published:** 2019-02-22

**Authors:** Haibin Yu, Wenyan Jia, Zhen Li, Feixiang Gong, Ding Yuan, Hong Zhang, Mingui Sun

**Affiliations:** 1 College of Electronics and Information, Hangzhou Dianzi University, Hangzhou, 310018 China; 2 Department of Electrical and Computer Engineering, University of Pittsburgh, Pittsburgh, USA; 3 Department of Computer Science, Ocean University of China, Qingdao, China; 4 Institute of Power Consumption and Energy Efficiency, China Electric Power Research Institute, Beijing, 100192 China; 5 Image Processing Center, School of Astronautics, Beihang University, Beijing, China; 6 Department of Neurological Surgery, University of Pittsburgh, Pittsburgh, USA

**Keywords:** Egocentric activity recognition, Activity of daily living, Multisource fusion, Knowledge-driven model, Dezert–Smarandache theory

## Abstract

Recently, egocentric activity recognition has attracted considerable attention in the pattern recognition and artificial intelligence communities because of its widespread applicability to human systems, including the evaluation of dietary and physical activity and the monitoring of patients and older adults. In this paper, we present a knowledge-driven multisource fusion framework for the recognition of egocentric activities in daily living (ADL). This framework employs Dezert–Smarandache theory across three information sources: the wearer’s knowledge, images acquired by a wearable camera, and sensor data from wearable inertial measurement units and GPS. A simple likelihood table is designed to provide routine ADL information for each individual. A well-trained convolutional neural network is then used to produce a set of textual tags that, along with routine information and other sensor data, are used to recognize ADLs based on information theory-based statistics and a support vector machine. Our experiments show that the proposed method accurately recognizes 15 predefined ADL classes, including a variety of sedentary activities that have previously been difficult to recognize. When applied to real-life data recorded using a self-constructed wearable device, our method outperforms previous approaches, and an average accuracy of 85.4% is achieved for the 15 ADLs.

## Introduction

In recent years, a variety of camera-based smart wearable devices have emerged in addition to smart watches and wristbands, such as Google Glass, Microsoft SenseCam, and Narrative. These wearables usually contain not only a camera, but also other sensors such as inertial measurement units (IMUs), global positioning system (GPS), temperature sensors, light sensors, barometers, and physiological sensors. These sensors automatically collect video/image, motion/orientation, environmental, and health data. Because these data are collected from the viewpoint of the wearer, they are called egocentric or first-person data. Tools for the automatic analysis and interpretation of egocentric data have been developed and applied to healthcare [1, 2], rehabilitation [3], smart homes/offices [4], sports [5], and security monitoring [6]. Egocentric activity recognition has now become a major topic of research in the fields of pattern recognition and artificial intelligence [7, 8].

Traditional methods of egocentric activity recognition often utilize motion sensor data from the IMU only and process these data using conventional classification techniques [9]. However, the performance of motion-based methods depends on the location of the IMU sensor on the body, and the classification accuracy tends to be lower when used to distinguish more complex activities in daily living (ADL), especially for certain sedentary activities. A wearable camera can provide more ADL information than motion sensors alone. Therefore, vision-based activity recognition using a wearable camera has become the focus of research in the field of egocentric activity recognition [10, 11].

In recent years, with the continuous development of the deep learning framework, the accuracy of image/video recognition has been improved greatly, and numerous vision-based activity recognition methods, such as deep learning, have emerged [12–14]. It has been reported that deep learning achieved a performance improvement of roughly 10% over the traditional trajectory tracking methods [14]. Although there has been significant progress in egocentric ADL recognition, the performance of vision-based methods is still subject to a number of constraints, such as the location of the wearable camera on the human body, image quality, variations in lighting conditions, occlusion, and illumination. In practical applications, no single sensor can be applied for all possible conditions. A common practice to avoid the risk of misrecognition by a single sensor is to fuse multiple recognition results for the same target from different sensors. Therefore, efforts have been made to combine vision and other sensor data for egocentric ADL recognition. For example, egocentric video and IMU data captured synchronously by Google Glass were used to recognize a number of ADL events [15]. Multiple streams of data were processed using convolutional neural networks (CNNs) and long- and short-term memory (LSTM), and the results were fused by maximum pooling. The average accuracy for 20 distinct ADLs reached 80.5%, whereas using individual video and sensor data only yielded accuracies of 75% and 49.5%, respectively. In [16], the dense trajectories of egocentric videos and temporally enhanced trajectory-like features of sensor data were extracted separately and then fused using the multimodal Fisher vector approach. The average recognition accuracy after fusion was 83.7%, compared to 78.4% for video-only and 69.0% for sensor-only data. These results show that, for egocentric ADL recognition, it is beneficial to integrate IMU sensors and cameras at both the hardware and algorithm levels.

Some commonly used multisource fusion methods include Bayesian reasoning, fuzzy-set reasoning, expert systems, and evidence theory composed of Dempster–Shafer evidence theory (DST) [17] and Dezert–Smarandache theory (DSmT) [18]. Among these methods, DST and DSmT have a simple form of reasoning and can represent imprecise and uncertain information using basic belief assignment functions, thus mimicking human thinking in uncertainty reasoning. By generalizing the discernment framework and proportionally redistributing the conflicting beliefs, DSmT usually outperforms DST when dealing with multisource fusion cases with conflicting evidence sources.

In egocentric ADL recognition using evidence theory, an activity model is often required to convert the activity data or features from different sources to the basic belief assignment (BBA). Generally, activity models can be divided into two types: data-driven and knowledge-driven [19]. Most ADLs have certain regularities because they occur at a relatively fixed time and place, and interact with a fixed combination of objects. As a result, abundant information about when, where, and how ADLs occur can be used to establish a knowledge base. Therefore, for ADL recognition, the knowledge-driven model is more intuitive and potentially powerful. Although no special knowledge-driven model for egocentric ADL recognition currently exists, some knowledge-driven models have been established in fields such as ADL recognition in smart homes, e.g., descriptive logic model [20], event calculus model [21], and activity ontology model [22]. Although these models offer semantic clarity and logical simplicity, they are usually complex. Users must contact the developers to convert their own daily routines into model parameters. Considering that this kind of model is best created by the wearers themselves, the current methods for knowledge representation require substantial simplification to improve their usability and adaptability for egocentric ADL recognition.

In this study, we propose a new knowledge-driven multisource fusion framework for egocentric ADL recognition and apply it to egocentric image sequences and other sensor data captured by a self-developed chest-worn device (eButton) [23] for diet and physical activity assessment. The main contributions of this study are as follows:

1. A knowledge-driven multisource fusion framework based on DSmT is established for the fusion of prior knowledge, vision-based results, and sensor-based results. This framework enables the accurate recognition of up to 15 kinds of ADLs, including a variety of sedentary activities that are hard to recognize using traditional motion-based methods, e.g., computer use, meetings, reading, telephone use, watching television, and writing.
2. The proposed knowledge-driven ADL model can be established by the device user. Previously, users were required to consult with an expert who could represent the user’s life experience quantitatively using certain index values. Our framework simplifies this process significantly, allowing individuals to express their ADL routines using a set of simple association tables.
3. A novel activity recognition algorithm based on egocentric images is proposed. With the help of “bags of tags” determined by CNN-based automatic image annotation, the complex image classification task is reduced to a text classification problem. Furthermore, the entropy-based term frequency-inverse document frequency (TF-IDF) algorithm is used to perform feature extraction and ADL recognition.

The remainder of this paper is organized as follows. Our methods for ADL recognition are described in detail in Section 2. A series of experimental results demonstrating the performance of the proposed framework are presented in Section 3. The comparison with existing methods is shown in Section 4. Finally, we conclude this paper in Section 5 by summarizing our approach and results and discussing some directions for future research.

## Methods

**Fig. 1:**
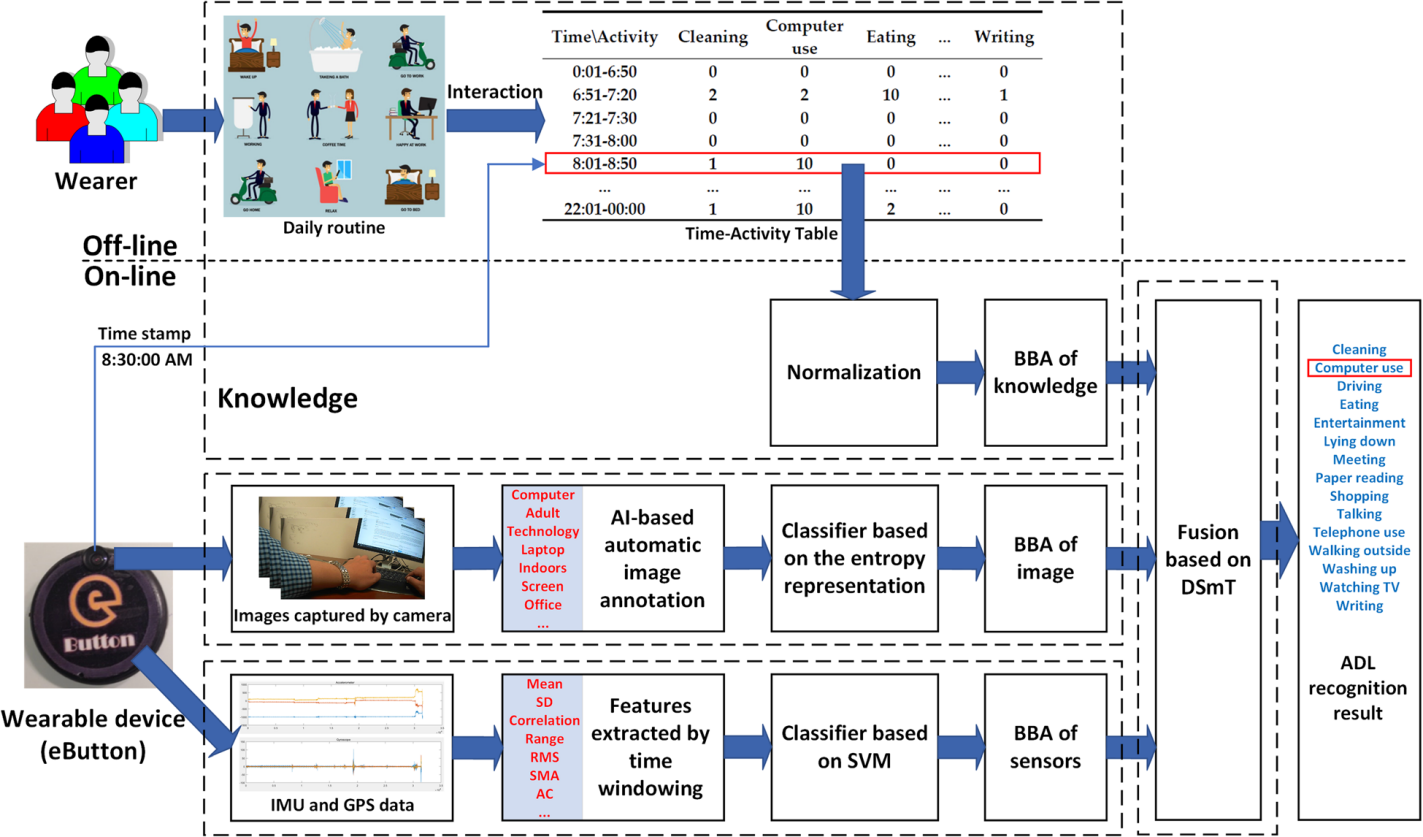
Architecture of the proposed method

Our multisource ADL recognition method is illustrated in Fig. 1. Conceptually, it consists of four main components: (1) basic information about the ADL routines of an individual (the user of the wearable device) is acquired using a “condition–activity” association table, (2) a CNN-based automatic image annotation pre-classifies the textual results using an entropy representation, (3) a set of motion and GPS data is processed and pre-classified using a support vector machine (SVM), and (4) a final classification is performed analytically by fusing the pre-classified results represented in terms of BBAs based on the DSmT framework.

### BBA of user knowledge

It is widely accepted that “the person who knows you the best is yourself,” although this is not universally true (e.g., a doctor may know better regarding illnesses). Nevertheless, people know their own lifestyle and ADL routines far better than other people or a computer. Therefore, we develop a knowledge-driven ADL model that can be established by the user of a wearable device. Previously, such a model would require the person to consult an expert who represents the user’s life experience quantitatively using certain index values [20–22]. In our framework, we simplify this process significantly to allow individuals to express their ADL routines using a set of simple association tables.

Let us consider *r* sources of information *ɛ*_1_, *ɛ*_2_, …, *ɛ*_*r*_. As each source may contain multiple information entities, each source *ɛ*_*i*_ is represented as a vector. With this definition, we represent pairwise relationships (*ɛ*_*i*_, *ɛ*_*j*_) from the *r* sources as a rectangular matrix. The matrix entry in row *ɛ*_*i*_ and column *ɛ*_*j*_ expresses the strength (a positive number) of the relation between these two elements. As the relationship between the two elements is not commutative, i.e., *A* leads to *B* does not imply *B* leads to *A*, the relationship matrix for (*ɛ*_*i*_, *ɛ*_*j*_) is generally asymmetric. As an important special case, (*ɛ*_*i*_, *ɛ*_*j*_) for *i* = *j* represents the relationships among the elements of *ɛ*_*i*_. According to Zintik and Zupan [24], all (*ɛ*_*i*_, *ɛ*_*j*_) can be tiled into a large, sparse global matrix.

As our knowledge-driven model runs under the framework of the Dezert–Smarandache theory, all activity-related conditions (e.g., time, place, order of occurrence) must be specified through the construction of numerical BBAs. Thus, if we view the ADLs and the conditions as different information sources, we can use the above theoretical framework to represent ADLs in relationships with certain conditions, including their time, place, and order of occurrence, and then fill the pairwise matrices (or tables) numerically. In our application, we require a simple and intuitive form that can be used by individuals. Therefore, we design each matrix as an association table containing integer values from 0 (impossible) to 10 (assured). For example, to represent one’s ADLs at different clock times, a hypothetical individual’s time–activity table is presented in Table 1. In this table, the wearer can adjust the time period according to his/her daily routine, especially activities with relatively clear start times, such as getting up, starting work, leaving work, and sleeping. Multiple time–activity tables may be required for weekdays and weekends/holidays (see the examples in Appendixes 1 and 2). Similarly, a location–activity table and an activity transition table (i.e., a table specifying the previous activity and the current activity) can be designed to further enrich the knowledge-driven model. Our experiments indicate that such tables can be completed quickly with little training.

**Table 1:** Sample time–activity table

Time period	Cleaning	Computer use	Eating	Entertainment	Lying down	Meeting	Reading	…*	Watching TV	Writing
0:01–6:50	0	0	0	0	10	0	0	…	0	0
6:51–7:20	2	2	10	0	0	0	3	…	0	1
7:21–7:30	0	0	0	0	0	0	0	…	0	0
…	…	…	…	…	…	…	…	…	…	…
21:01–22:00	2	10	1	0	0	0	3	…	9	2
22:01–00:00	1	10	2	0	5	0	0	…	9	0

*Six columns (indicated by “…”) are omitted in the table, namely “shopping,” “talking,” “telephone use,” “transportation,” “walking outside,” and “washing up”

Considering that the BBA value for each activity should be between 0 and 1 (see Section 2.4), we apply row-wise normalization according to the sum of all integer values in that row. For the example in Table 2, if the clock time is 21:18:00, the corresponding BBA is constructed by dividing all integer values in the “21:01–22:00” row by the sum of these values.

**Table 2:** BBA values of the user-provided knowledge of ADLs, based on Table 1 and a time stamp of 21:18:00

Time period	Cleaning	Computer use	Eating	Entertainment	Lying down	Meeting	Reading	…*	Watching TV	Writing
21:01–22:00	0.0488	0.2439	0.0244	0	0	0	0.0732	…	0.2195	0.0488

*Six columns (indicated by “…”) are omitted in the table, namely “shopping,” “talking,” “telephone use,” “transportation,” “walking outside,” and “washing up”

### BBA of images

In our case, activity recognition from egocentric images must be performed indirectly, because the person wearing the camera is unlikely to appear in the images. We perform the recognition task using the concept of a combination of objects (CoO) [25, 26]. For example, “computer use” is likely to have a CoO consisting of a computer, monitor, screen, keyboard, and table. When this CoO is fully or partially observed, the underlying activity can be guessed with a certain degree of confidence. In this study, the two main steps for ADL recognition using the CoO concept are (1) extraction of CoO and (2) construction of an ADL classifier. These steps are detailed below.

#### Semantic feature extraction by CNN

In this study, we are mainly concerned with whether ADL-related objects are present in the input image, rather than their order of presentation (although the order may also carry some information). Ignoring the order, we perform the CoO detection task in two steps. In the first step, all objects in the input image are detected and represented in the form of a textual list. This is essentially a process of automatic image annotation. In the second step, we check whether there is a CoO corresponding to a particular ADL in the list.

**Fig. 2:**
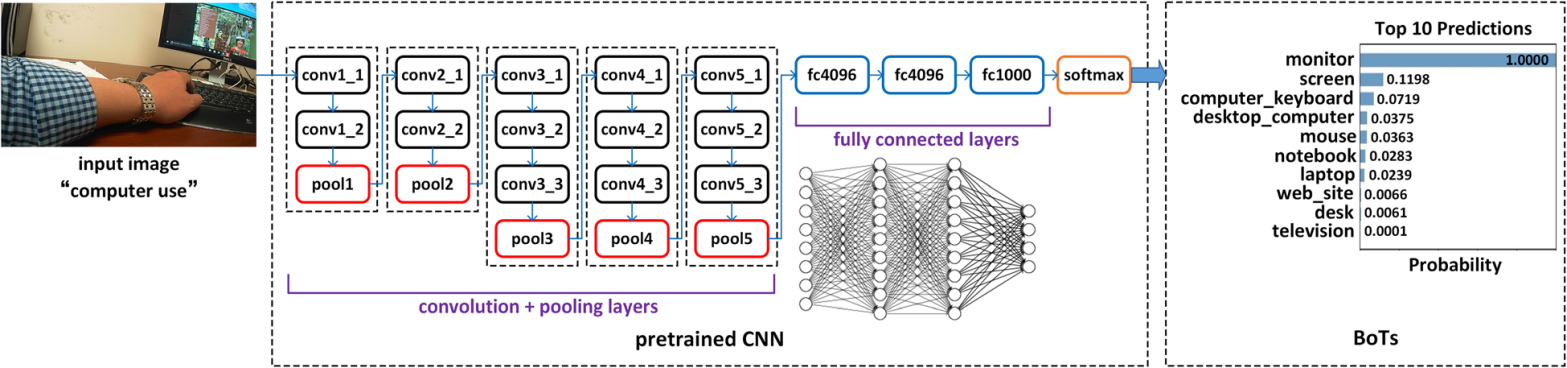
The typical process of automatic image classification and annotation using pre-trained CNN

Recently, with the continuous development of the deep learning framework, automatic image annotation can produce impressive image annotation results with the aid of well-trained CNNs. A CNN is a class of deep, feed-forward artificial neural networks that generally include a convolutional layer, a pooling layer, and a fully connected layer. Some well-known pre-trained CNNs include AlexNet [27], VGGNet [28], and ClarifaiNet [29, 30] which are pre-trained using a large image database such as ImageNet [31]. The typical process of automatic image classification and annotation using the pre-trained CNN is shown in Fig. 2 (considering the VGG-16 network in VGGNet as an example). The output of the automatic image annotation is a series of textual tags, which can be defined as “bag of tags” (BoTs). As the BoTs are extracted from a specific image, it can be regarded as the high-level semantic feature of the image.

**Fig. 3:**
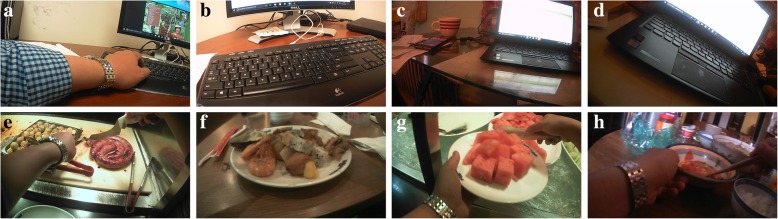
Examples of egocentric images of different activities. **a**–**d** are the egocentric images of “computer use”; **e**–**h** are the egocentric images of “eating”

After comparison, we find that the textual tags extracted by ClarifaiNet are more consistent with the objects in the images of our egocentric dataset. Therefore, we use ClarifaiNet and adopt a process exemplified in Fig. 2 to obtain the BoTs of each frame in the egocentric image sequence, i.e.,

1 $$ {\mathrm{BoTs}}_i={\mathrm{CNN}}_{\mathrm{ClarifaiNet}}\left({I}_i\right)=\left\{{T}_1^i,{T}_2^i,\dots, {T}_L^i\right\} $$

where *I*_*i*_ is the *i*th frame in the image sequence, *T* is the extracted tag, and *L* is the number of tags extracted from one frame of the image (when using ClarifaiNet, the default value of *L* is 20). An example of BoTs is shown in Table 3, and the images corresponding to these BoTs are shown in Fig. 3.

**Table 3:** BoTs produced by ClarifaiNet for the egocentric images in Fig. 3

Image no.	BoTs							
	1	2	3	4	5	6	7	…
a	Computer	Technology	Business	Laptop	People	Indoors	Keyboard	…
b	Computer	Technology	Keyboard	Internet	Laptop	Business	Electronics	…
c	Room	No person	Table	Business	Computer	Indoors	Office	…
d	Computer	Technology	Laptop	Internet	No person	Business	Keyboard	…
e	Food	People	Knife	Indoors	Meat	Restaurant	Cooking	
f	Food	No person	Meat	Fish	Dinner	Meal	Plate	…
g	Food	Indoors	People	Knife	Sugar	Fruit	Cooking	…
h	People	Indoors	Container	Drink	Food	Table	Tableware	…

#### BBA construction from BoTs

As mentioned above, CNN-produced BoTs can be regarded as a high-level semantic feature from the specific egocentric image. Hence, it can be used in the classification of the ADL corresponding to the image. For example, the tags in Table 3 correspond to two ADLs, “computer use” and “eating.” We can select certain keywords to represent these activities, e.g., “computer use” can be represented by the set {“computer,” “technology,” “laptop,” “keyboard,” “internet”} and “eating” corresponds to the set {“food,” “meat,” “cooking,” “plate”}. Table 3 also indicates that both sets contain some less general or non-distinctive tags such as “no person,” “people,” and “indoors.” Moreover, there may be substantial differences among the tags extracted from the same activity class because of different image contexts and acquisition parameters (e.g., distance, view angle). Therefore, the classification accuracy depends on selecting tags that not only describe the target activity within a class, but also distinguish activities across classes.

With the BoTs constructed in this way, ADL recognition from egocentric images becomes a semantic textual classification task. We approach this task using the vector space model [32] to represent BoTs and establish a text classifier. First, we compute the term frequency-inverse document frequency (TF-IDF) measure, which is widely used for weighting textual features, given by [33]

2 $$ {tf}_{i,j}\cdot {idf}_i=\frac{n_{i,j}}{\sum_k{n}_{k,j}}\cdot \log \frac{\left|D\right|}{\left|\left\{j:{t}_i\in {d}_j,{d}_j\in D\right\}\right|+1} $$

where *tf*_*i*, *j*_ and *n*_*i*, *j*_ denote the term frequency and number of occurrences of *t*_*i*_ in document *d*_*j*_, respectively; ∑_*k*_*n*_*k*, *j*_ is the sum of the occurrences of all terms in document *d*_*j*_ (i.e., the total number of terms); *idf*_*i*_ is the inverse document frequency (a measure of whether the term is common or rare across all documents) of term *t*_*i*_; |{*j* : *t*_*i*_ ∈ *d*_*j*_,  *d*_*j*_ ∈ *D*}| is the number of documents containing term *t*_*i*_ in document set *D*; and |*D*| is the number of documents in *D*. Note that (2) does not apply to the case where the document set contains different types of documents, i.e., it cannot be used directly to classify a BoT set containing different ADLs. To apply TF-IDF to document sets containing multiple types of documents, a number of modified algorithms have been developed, including bidirectional normalization for the term frequency [34], constraints imposed by the mutual information [35], and the application of information entropy [36]. The entropy-based TF-IDF generally provides better classification because the statistical features of the terms among different types of documents can be well-represented by the information entropy. We modify the entropy approach by adding an inter-class entropy factor *e*1_*i*, *k*_ and an intra-class entropy factor *e*2_*i*_ to (2). This allows the BoT classifier to “compact” the intra-class activities while “separating” inter-class activities, as described below.

Assuming that the total number of the ADLs to be classified is *K*, the corresponding egocentric image set is *A* = {*A*_1_, *A*_2_,  … , *A*_*K*_}. For the *k*th activity *A*_*k*_ ∈ *A*, the total number of images is |*A*_*k*_| and all BoTs extracted from each image in *A*_*k*_ constitute the BoT subset ${B}_{A_k}=\left\{{B}_1,{B}_2,\dots, {B}_{\left|{A}_k\right|}\right\}$. For the BoT set of *A*, we then have ${B}_A=\left\{{B}_{A_1},{B}_{A_2},\dots, {B}_{A_k},\dots, {B}_{A_{K-1}},{B}_{A_K}\right\}$ with $\left|A\right|=\sum \limits_{k=1}^K\left|{A}_k\right|$. Assume that there are *N* unique tags *T* = {*T*_1_, *T*_2_,  … , *T*_*N*_} in *B*_*A*_. For any tag *T*_*i*_ ∈ *T*, its inter-class entropy factor for ${B}_{A_k}$, called *e*1_*i*, *k*_, can be defined as

3 $$ e{1}_{i,k}=-\sum \limits_{j=1}^{\left|{A}_k\right|}\frac{C\left({B}_j,{T}_i\right)}{C\left({B}_{A_k},{T}_i\right)}\cdot {\log}_2\frac{C\left({B}_j,{T}_i\right)}{C\left({B}_{A_k},{T}_i\right)} $$

where *C*(*B*_*j*_, *T*_*i*_) is the number of occurrences of tag *T*_*i*_ in *B*_*j*_ (i.e., the *j*th subset of ${B}_{A_k}$), given by

4 $$ C\left({B}_j,{T}_i\right)={\sum}_l\left[{T}_i=={B}_j(l)\right],\kern0.5em {B}_j\in {B}_{A_k}, $$

where the double equation signs denote “whether the two operands are equal,” resulting in a binary output for the bracketed variable. Using (4), $C\left({B}_{A_k},{T}_i\right)$ can be expressed as

5 $$ C\left({B}_{A_k},{T}_i\right)=\sum \limits_{j=1}^{\left|{A}_k\right|}C\left({B}_j,{T}_i\right). $$

The intra-class entropy of *T*_*i*_ for *B*_*A*_, called *e*2_*i*_, can be defined as

6 $$ e{2}_i=-\sum \limits_{k=1}^K\frac{D\left({B}_{A_k},{T}_i\right)}{D\left({B}_A,{T}_i\right)}\cdot {\log}_2\frac{D\left({B}_{A_k},{T}_i\right)}{D\left({B}_A,{T}_i\right)} $$

where $D\left({B}_{A_k},{T}_i\right)$ is the number of BoTs containing tag *T*_*i*_ in subset ${B}_{A_k}$, defined as

7 $$ D\left({B}_{A_k},{T}_i\right)=\left|\left\{j:{T}_i\in {B}_j,\kern1em {B}_j\in {B}_{A_k}\right\}\right|. $$

From this definition of $D\left({B}_{A_k},{T}_i\right)$, we can express *D*(*B*_*A*_, *T*_*i*_) as

8 $$ D\left({B}_A,{T}_i\right)=\sum \limits_{k=1}^KD\left({B}_{A_k},{T}_i\right)=\sum \limits_{k=1}^K\left|\left\{j:{T}_i\in {B}_j,\kern1em {B}_j\in {B}_{A_k}\right\}\right|. $$

It can be observed from (3) that *e*1_*i*, *k*_ is used to describe the distribution of tag *T*_*i*_ in ${B}_{A_k}$, which corresponds to the particular activity *A*_*k*_. Moreover, the more uniform the distribution of *T*_*i*_ in ${B}_{A_k}$, the larger the value of *e*1_*i*, *k*_ and, consequently, the greater the contribution of the *T*_*i*_ to the classification of activity *A*_*k*_. Similarly, in (6), *e*2_*i*_ is used to describe the distribution of tag *T*_*i*_ across the BoT subsets in *B*_*A*_, which corresponds to all different activities. When *e*2_*i*_ reaches its maximum, however, the *T*_*i*_ are uniformly distributed among the BoT subsets in *B*_*A*_, which means that *T*_*i*_ has no ability to distinguish different activities. Therefore, the value of *e*2_*i*_ is inversely proportional to its contribution to the classification, which is the opposite of *e*1_*i*, *k*_. Balancing these two effects, the entropy-based TF-IDF is given by

9 $$ {tf}_{i,k}\cdot {idf}_i\cdot e{1}_{i,k}\cdot R\left(e{2}_i\right)={tf}_{i,k}\cdot {idf}_i\cdot e{1}_{i,k}\cdot \left(1-\frac{e{2}_i}{\log_2K+\lambda}\right) $$

where *R*(*e*2_*i*_) = 1 − *e*2_*i*_/(log_2_*K* + *λ*) is used to remap *e*2_*i*_ so that its value is proportional to the contribution in the classification. The parameter *λ* is an empirically determined small positive constant that guarantees *R*(*e*2_*i*_) > 0.

Using (9), the BoT classifier can be obtained by applying a suitable training procedure. Specifically, the entropy-based TF-IDF weight of each tag in the sample BoT set is calculated, and the *M* tags with the highest weight values are extracted from ${B}_{A_k}$ to form the class center vector *ζ*_*k*_ corresponding to activity *A*_*k*_. All class center vectors constitute the BoT classifier, given by

10 $$ {\mathrm{Classifier}}_B=\left\{{\zeta}_1,{\zeta}_2,\dots, {\zeta}_k,\dots, {\zeta}_K\right\}. $$

An example of the BoT classifier is presented in Table 4.

**Table 4:** Example of the BoT classifier

Activity	*ζ*_*k*_ with the entropy-based TF-IDF value						
	1	2	3	4	5	6	…
Computer use	Keyboard	Monitor	Screen	Internet	Electronics	Laptop	…
	0.4328	0.3792	0.3255	0.3127	0.3071	0.2662	…
Eating	Food	Drink	Restaurant	Dinner	Cooking	Bowl	…
	0.3678	0.3286	0.3240	0.2894	0.2594	0.2361	…
Shopping	Stock	Market	Shopping	Shop	Merchandise	Supermarket	…
	0.4216	0.4185	0.4079	0.3363	0.2724	0.2373	…
Washing up	Bathroom	Wash	Bath	Hygiene	Faucet	Bathtub	…
	0.4955	0.4375	0.2879	0.2859	0.2789	0.2699	
Transportation	Dashboard	Steering wheel	Control	Fast	Drive	Driver	…
	0.2769	0.2769	0.2733	0.2716	0.2696	0.2669	
…	…	…	…	…	…	…	…

When using the classifier defined in (10), the cosine similarity between the input BoT and the center vector of each class (i.e., *ζ*_*k*_) can be calculated, and the class whose center is closest to the input is assigned as the classification result. In addition, as the cosine similarity is between 0 and 1, it can be directly used to form the BBA for images; an example of this can be seen in the third row (BBA of image) of Table 6.

### BBA of IMU and GPS sensors

For IMU sensors, the output data are multiple 1-D waveforms that can be processed using traditional pattern recognition methods [9]. First, the data are divided into non-overlapping segments, and the structural and statistical features of each segment are extracted. These features are used to train a classifier. The training ends when a certain stopping criterion is met.

IMU sensors include an accelerometer and a gyroscope, each producing three traces of signals in the *x*-, *y*-, and *z*-axes. These signals are divided into 3-s segments without overlapping. To synchronize them with the corresponding images, each segment is centered around the time stamp in the image data. The features extracted in each segment include the mean, standard deviation, correlation, signal range (difference between maximum and minimum), root mean square, signal magnitude area [37], autoregressive coefficients (calculated up to the sixth order), and the binned distribution (selected to be 10) [38]. These features are combined with the GPS velocity and coordinates (if unavailable, the most recent GPS data are used) to form 127-dimentional feature vectors that are fed into a multiclass SVM for training and classification.

Support vector machine (SVM) [39] is a supervised machine learning method widely used in classification and regression analysis. SVM can improve the generalization ability of a learning machine by minimizing the structural risk; hence, it can also yield reasonably good statistical rules for a relatively small sample size. The dual objective function of SVM can be given by the Lagrangian multiplier method as shown below

11 $$ \underset{\alpha_i\ge 0}{\max}\underset{w,b}{\min}\mathcal{L}\left(w,b,\alpha \right)=\underset{\alpha_i\ge 0}{\max}\underset{w,b}{\min}\left(\frac{1}{2}{\left\Vert w\right\Vert}^2-\sum \limits_{i=1}^n{\alpha}_i\left({y}_i\left({w}^T{x}_i+b\right)-1\right)\right) $$

where *x* is the input data, *y* is the category to which *x* belongs, *w* is the vector perpendicular to the classification hyperplane, *b* is the intercept, and *α* is the Lagrange multiplier.

After solving (11) using the quadratic programming algorithm and introducing the kernel function *κ*(*x*_1_, *x*_2_) = (〈*x*_1_, *x*_2_〉 + 1)^2^ to map the data to the high-dimensional space, SVM can perform a nonlinear classification according to the following binary prediction:

12 $$ {g}_{\mathrm{SVM}}(x)=\operatorname{sign}\left({w}^Tx+b\right)=\operatorname{sign}\left(\sum \limits_{i=1}^N{\alpha}_i{y}_i\kappa \left({x}_i,\kern0.5em x\right)+b\right). $$

Commonly used kernel functions include polynomial kernel and radial basis function.

The SVM is fundamentally a two-class classifier; however, it can be extended to multiclass problems by using one-against-one or one-against-all voting schemes. In addition, the basic SVM classifier can only output the classification label rather than the probability or possibility for evidence fusion. To solve this problem, the “libsvm” [40] toolkit, which converts the output of the standard SVM to a posterior probability using a sigmoid-fitting method [41], is utilized. An example is provided in the fourth row (BBA of sensors) of Table 6.

### Hierarchical fusion of knowledge, image, and sensor data by DSmT

In DSmT, the discernment framework Θ = {*θ*_1_, *θ*_2_,  … , *θ*_*n*_} is extended from the power set 2^Θ^ in Dempster–Shafer theory to the hyper-power set. The hyper-power set, denoted by *D*^Θ^, admits the intersections of elements on the basis of the power set. For example, if there are two elements in the discernment framework Θ = {*θ*_1_, *θ*_2_}, the power set is 2^Θ^ = {∅, *θ*_1_, *θ*_2_, *θ*_1_ ∪ *θ*_2_} and the hyper-power set is *D*^Θ^ = {∅, *θ*_1_, *θ*_2_, *θ*_1_ ∪ *θ*_2_, *θ*_1_ ∩ *θ*_2_}. The BBA defined on the hyper-power set *D*^Θ^ is

13 $$ \left\{\begin{array}{l}m\left({X}_i\right):{D}^{\Theta}\to \left[0,1\right],\kern1em {X}_i\in {D}^{\Theta}\\ {}m\left(\varnothing \right)=0,\kern1em \sum \limits_{\theta \in {D}^{\Theta}}m\left(\theta \right)=1\end{array}\right. $$

The combination rule is the core of evidence theory. It combines the BBAs of different sources within the same discernment framework to produce a new belief assignment as the output. In the DSmT framework, the most widely used combination rule is the Proportional Conflict Redistribution (PCR) rule. There are six PCR rules (PCR1–PCR6), defined in [18]. Their differences are mainly in the method of proportional redistribution of the conflicting beliefs. Among these rules, PCR5 is widely used to combine two sources and PCR6 is usually applied to more than two sources. In particular, PCR6 is the same as PCR5 when there are exactly two sources. If *s* represents the number of sources, the PCR5/PCR6 combination rule for *s* = 2 is

14 $$ {\displaystyle \begin{array}{l}{m}_{1\oplus 2}^{PCR5/ PCR6}(A)=\\ {}\sum \limits_{\begin{array}{l}{X}_1,{X}_2\in {D}^{\Theta}\\ {}{X}_1\cap {X}_2=A\end{array}}{m}_1\left({X}_1\right){m}_2\left({X}_2\right)+\sum \limits_{\begin{array}{l}X\in {D}^{\Theta}\\ {}X\cap A=\varnothing \end{array}}\left[\frac{m_1^2(A){m}_2(X)}{m_1(A)+{m}_2(X)}+\frac{m_2^2(A){m}_1(X)}{m_2(A)+{m}_1(X)}\right]\end{array}} $$

where *m*_1 ⊕ 2_ denotes *m*_1_ ⊕ *m*_2_, i.e., sources 1 and 2 are used for evidence fusion for the focal element *A* in discernment framework *D*^Θ^. The PCR6 combination rule for *s* > 2 is

15 $$ {\displaystyle \begin{array}{l}{m}_{1\oplus 2\oplus \dots \oplus s}^{PCR6}(A)=\sum \limits_{\begin{array}{l}{X}_1,{X}_2,\dots, {X}_s\in {D}^{\Theta}\\ {}\kern1.5em {\cap}_{i=1}^s{X}_i=A\end{array}}\prod \limits_{i=1}^s{m}_i\left({X}_i\right)+\\ {}\sum \limits_{\begin{array}{l}{X}_1,{X}_2,\dots, {X}_{s-1}\in {D}^{\Theta}\\ {}{X}_i\ne A,i\in \left\{1,2,\dots, s-1\right\}\\ {}\kern1em \left({\cap}_{j=1}^{s-1}{X}_i\right)\cap A=\varnothing \end{array}}\sum \limits_{k=1}^{s-1}\sum \limits_{\left({i}_1,{i}_2,\dots, {i}_s\right)\in P\left(1,2,\dots, s\right)}\left[\sum \limits_{p=1}^k{m}_{i_p}(A)\cdot \frac{\prod \limits_{j=1}^k{m}_{i_j}(A)\prod \limits_{p=k+1}^{s-1}{m}_{i_p}\left({X}_p\right)}{\sum \limits_{j=1}^k{m}_{i_j}(A)+\sum \limits_{p=k+1}^{s-1}{m}_{i_p}\left({X}_p\right)}\right]\end{array}} $$

where *P*(1,  … , *s*) is the set of all permutations of elements {1,  … , *s*}.

In the proposed approach, when DSmT is used for ADL recognition, the discernment framework contains 15 ADLs, as detailed in Eq. (16) and Table 5.

16 $$ {\displaystyle \begin{array}{l}\Theta =\left\{{A}_1,{A}_2,\dots, {A}_{15}\right\}=\\ {}\Big\{{}^{``}\mathrm{cleaning},{}^{"``}\mathrm{computer}\kern0.5em \mathrm{use},{}^{"``}\mathrm{eating},{}^{"``}\mathrm{entertainment},{}^{"}\kern0.5em \\ {}{\kern0.24em }^{``}\mathrm{lying}\kern0.5em \mathrm{down},{}^{"``}\mathrm{meeting},{}^{"``}\mathrm{reading},{}^{"``}\mathrm{shopping},{}^{"``}\mathrm{talking},{}^{"}\\ {}{\kern0.24em }^{``}\mathrm{telephone}\kern0.5em \mathrm{use},{}^{"``}\mathrm{transportation},{}^{"``}\mathrm{walking}\kern0.5em \mathrm{outside},{}^{"}\kern0.5em \\ {}{\kern0.24em }^{``}\mathrm{washing}\kern0.5em \mathrm{up},{}^{"``}\mathrm{watching}\kern0.5em \mathrm{TV},{}^{"``}{\mathrm{writing}}^{"}\Big\}\kern0.86em \end{array}} $$

**Table 5:** The description of the discernment framework defined in Eq. (16)

Θ					
1	Cleaning (CN)	6	Meeting (MT)	11	Transportation (TP)
2	Computer use (CU)	7	Reading (RD)	12	Walking outside (WO)
3	Eating (ET)	8	Shopping (SP)	13	Washing up (WU)
4	Entertainment (EM)	9	Talking (TK)	14	Watching TV (TV)
5	Lying down (LD)	10	Telephone use (TU)	15	Writing (WT)

As the total number of sources is three (i.e., knowledge, image, and sensor data), PCR6 should be selected as the evidence combination rule if all sources are used in the data fusion process. An example of the fusion result from three sources using (15) is presented in Table 6. In this example, the BBAs of knowledge, image, and sensor data are derived from the time–activity table, cosine similarity between current BoT and class center, and posterior probability of the support vector machine classifier’s output, respectively.

**Table 6:** Example of three-source fusion using the PCR6 rule

	CN	CU	ET	EM	LD	MT	RD	SP	TK	TU	TP	WO	WU	TV	WT
BBA of knowledge	0.1860	0.0233	0.2326	0.1163	0	0	0.0233	0	0.1163	0.1860	0	0.0698	0.0233	0.0233	0
BBA of image	0.0401	0.0260	0	*0.4452*	0	0.1526	0.0610	0	0.0939	0.1505	0	0	0	0	0.0308
BBA of sensors	0.0041	0.0303	0.0078	0.0558	0.0338	0.0076	0.0077	0.0229	0.1781	0.0264	0.0101	0.0174	0.0178	*0.5602*	0.0200
Fusion result	0.0565	0.0057	0.0754	*0.2561*	0.0031	0.0427	0.0103	0.0015	0.1017	0.0960	0.0003	0.0106	0.0022	*0.3341*	0.0037

Conditions: the time stamp of the camera is 17:30:57 on Thursday. The captured image can be seen in Fig. 6(d), and the ground truth is “entertainment”

Italics represent the maximum value of the BBA for all activities of the same information source, and the corresponding activity is the recognition result of that information source

In our case, the information sources differ greatly in the signal type and processing algorithm, e.g., the image source provides a specific combination of objects, whereas the sensor source provides the motion status of the person wearing the device. Hence, the corresponding recognition results are often different. This can be observed in Table 6. For the same activity, the recognition results from the image and sensor sources are “entertainment” and “watching TV,” respectively. In fact, “entertainment” (specifically “playing poker” in this case) and “watching TV” are both sedentary activities, and it is difficult to distinguish them using motion sensors (both the IMU and GPS sensors). Therefore, the recognition result from the image source should be more reliable. However, after fusion, the final recognition result is “watching TV” because the belief value of “entertainment” assigned by the BBA of the sensors is very low.

Based on previous research [15, 16] and our own study (described in Section 3), most ADLs achieve significantly higher accuracy when using vision-based data than with motion sensor-based data. Thus, in many cases, if the three sources of information are fused directly, the accuracy of the output is often affected by the low specificity of the motion sensors. However, we still need to use motion sensors to identify ADLs that have significant motion signatures, such as “cleaning,” “walking outside,” and “lying down.” Therefore, considering the reliability of each information source, we consider user knowledge and image sources to be high-priority data and the motion sensor source to be low-priority data, i.e., we supplement the sensor information only when the fusion of user knowledge and image sources leads to a conflict.

**Fig. 4:**
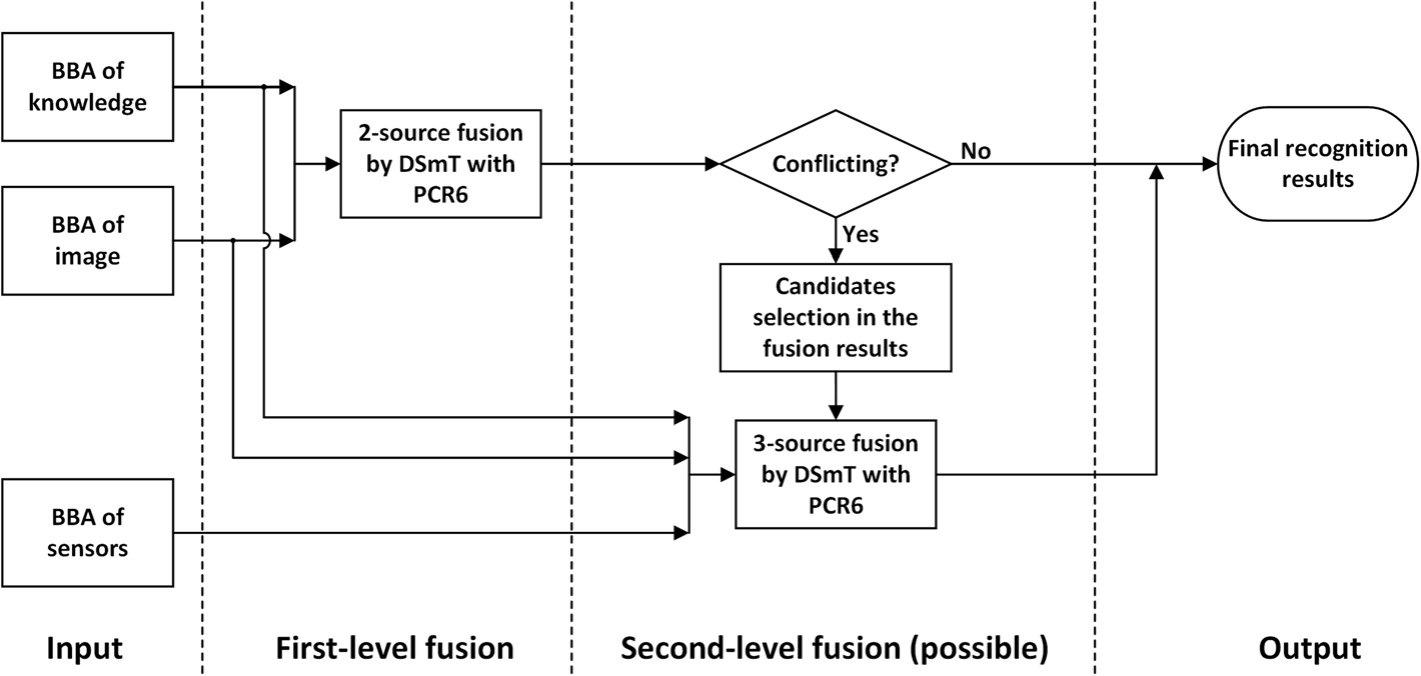
Architecture of 2-L HFNDCS

We implement the source-priority concept using a two-level hierarchical fusion network with descending candidate sets (2-L HFNDCS, see Fig. 4), similar to the implementation strategy proposed in [42, 43]. When the two-source fusion between the knowledge and image-based methods provides a conflicting result, motion sensor data are added to the pool of evidence for a second-level three-source fusion. Instead of considering all activities, only the candidate activities identified by two-source fusion are used as the input for the three-source fusion. The initial number of candidate activities is given in advance, and this number can be adjusted according to subsequent test results. The output of the final fusion is the activity with the highest belief among the candidate activities. The 2-L HFNDCS algorithm can be described as follows.

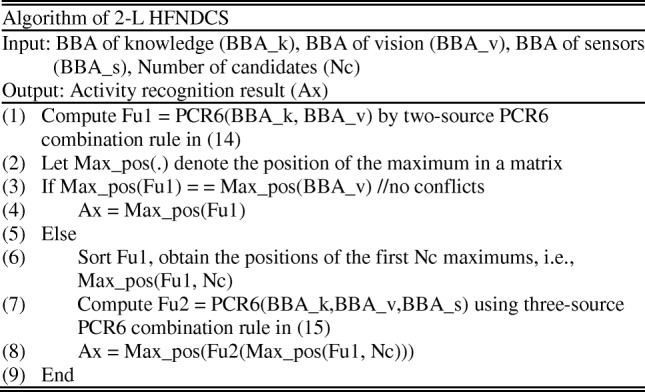



## Experimental results

### Experimental setup and data acquisition

**Fig. 5:**
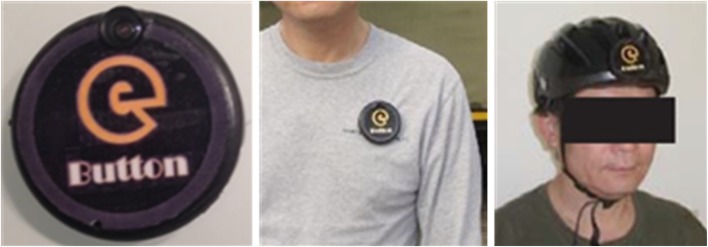
Appearance of the eButton and examples of its wearing methods

Previously, our laboratory developed eButton (Fig. 5), a disk-like wearable device the size of an Oreo cookie that can be used to study human diet, physical activity, and sedentary behavior [23]. The eButton is equipped with a camera, IMU, and other sensors that are not used for the current study, such as those for measuring the temperature, lighting, and atmospheric pressure. The resolution of the camera is 1280 × 720 pixels. To save power, the camera acquires one image every 4 s. The built-in IMU contains a three-axis accelerometer and a three-axis gyroscope with a sampling frequency of 90 Hz. The GPS data are acquired from the wearer’s mobile phone at 1-s intervals and synchronized with the eButton data using time stamps.

Two volunteers with regular daily routines and relatively invariant living environments were selected for our experiments. After signing a consent form approved by the Institutional Review Board, they were asked to fill out the time–activity table described above. Their time–activity tables are provided in Appendixes 1 and 2. The volunteers then wore the eButton for a relatively long time (approximately 10 h per day for about 3 months). To form a gold standard for performance comparison, the resulting egocentric data were manually reviewed and annotated. For regular daily routines, the environment and motion patterns corresponding to certain activities were very similar. In contrast, the frequency and duration vary widely among less regular activities, resulting in a large imbalance among the number of samples corresponding to different activities. To reduce this data imbalance, a key frame extraction method was used [44, 45]. As the two eButton wearers each participated in the study for about 3 months, we had sufficient data to form two independent datasets (one for training and one for testing). We combined these data to form an egocentric activity dataset, called the eButton activity dataset [47].

**Table 7:** Numbers of key frames in the image subset

Set		CN	CU	ET	EM	LD	MT	RD	SP	TK	TU	TP*	WO	WU	TV**	WT
Training set	W1	127	139	115	117	153	99	146	170	79	106	185	101	102	84	97
	W2	123	119	149	105	120	84	112	107	80	95	106	97	113	–	108
Test set	W1	113	139	155	59	178	92	101	149	70	99	184	146	90	70	125
	W2	120	95	159	87	197	95	91	91	42	98	128	98	95	–	109

*Transportation method differs between the two wearers; W1 drives and W2 uses the bus

**W2 does not watch TV

**Fig. 6:**
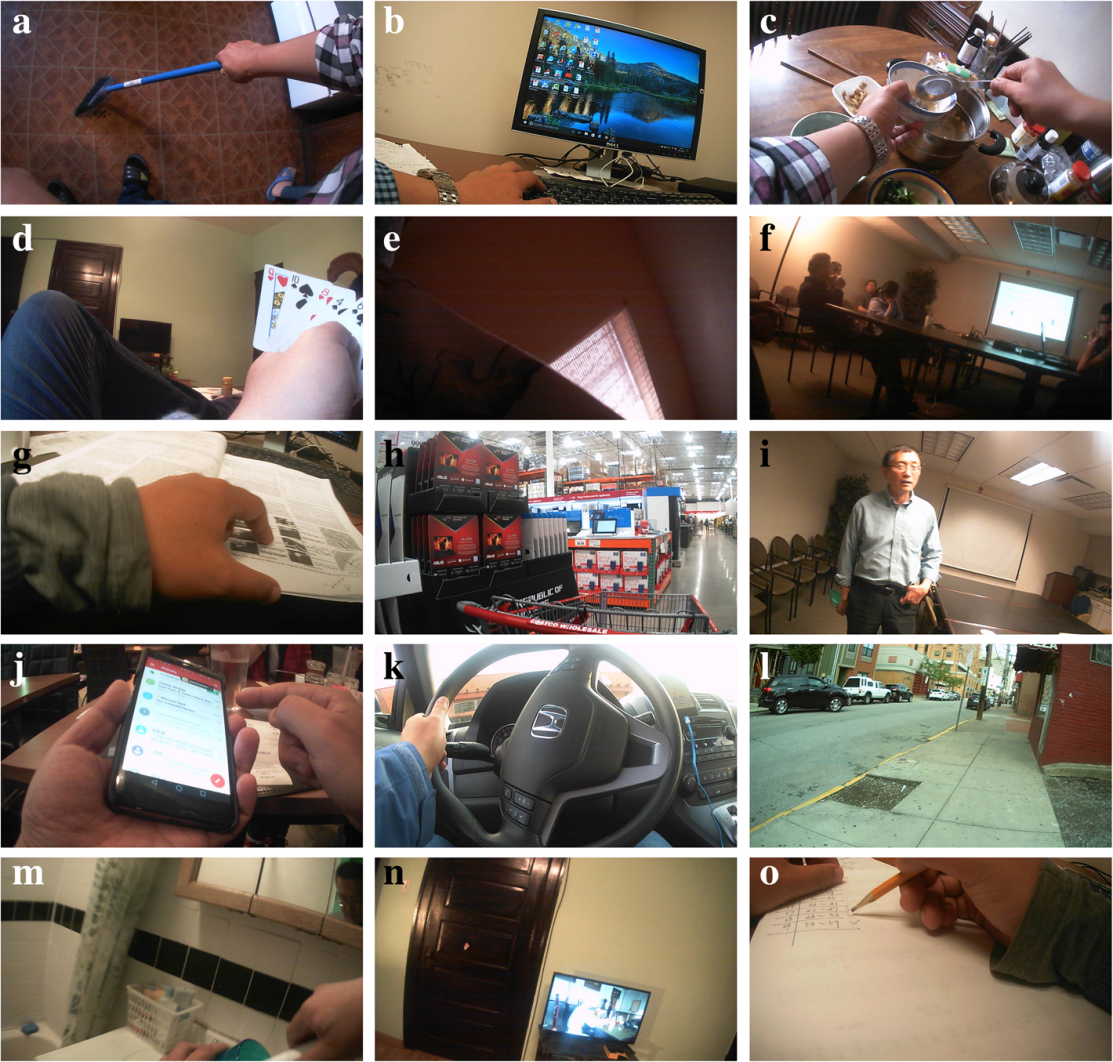
The sample image of each activity in the training set. Images **a** through **o** correspond to “cleaning,” “computer use,” “eating,” “entertainment,” “lying down,” “meeting,” “reading,” “shopping,” “talking,” “telephone use,” “transportation” (driving), “walking outside,” “washing up,” “watching TV,” and “writing,” respectively

In the eButton activity dataset, each wearer (referred to as W1 and W2) has a separate set of time–activity tables, a training set, and a test set. Although the training set and the test set do not overlap, they both have the same structure: a subset of egocentric images, a subset of motion sensor data, and a GPS data file. In the subset of egocentric images, each activity to be recognized corresponds to an image sequence. Each frame in the image sequence was extracted by the key frame extraction method [44, 45]. The number of key frames corresponding to different activities is listed in Table 7, and some sample frames are shown in Fig. 6. The file name of each key frame includes the specific time stamp. In the motion sensor subset, there is a one-to-one correspondence between the motion sensor data and the images in the image subset, i.e., each image corresponds to a motion sensor data file. The motion sensor files contain all raw sensor data (three-axis acceleration and three-axis gyroscope) from within a 3-s window centered around the stamp time of the image. There is also a one-to-one correspondence between the GPS data and the image subset. The GPS data (including time, coordinates, velocity, etc.) are synchronized with the time stamp of an image and recorded in one row of the GPS data file.

**Fig. 7:**
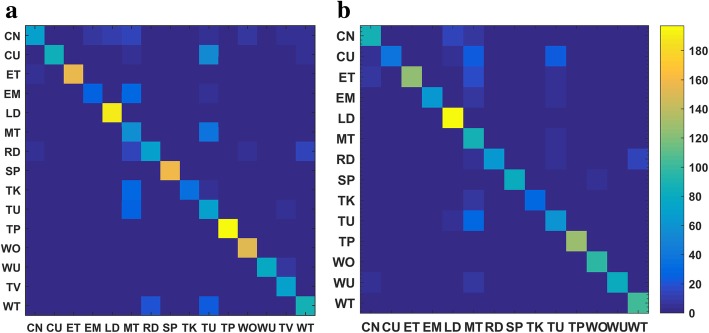
Confusion matrices for the image-based method as applied to **a** wearer 1 and **b** wearer 2

### Experimental results

All data were analyzed using Matlab 8.6 on a PC running Windows 10 Pro. To facilitate the performance evaluation and comparison, the *F*_1_ measure [46], which is commonly used in the field of pattern recognition, was selected as the criterion for evaluating different classification methods. *F*_1_ is defined as

17 $$ {\displaystyle \begin{array}{l}{F}_1=2\cdot PR/\left(P+R\right)\\ {}P= TP/\left( TP+ FP\right),\kern1em R= TP/\left( TP+ FN\right)\end{array}} $$

where *P* is precision and *R* is recall. *TP*, *FP*, and *FN* represent the number of true samples, false positive samples, and false negative samples, respectively, derived from the confusion matrix. *F*_1_ is also called the harmonic mean of recall and precision.

**Fig. 8:**
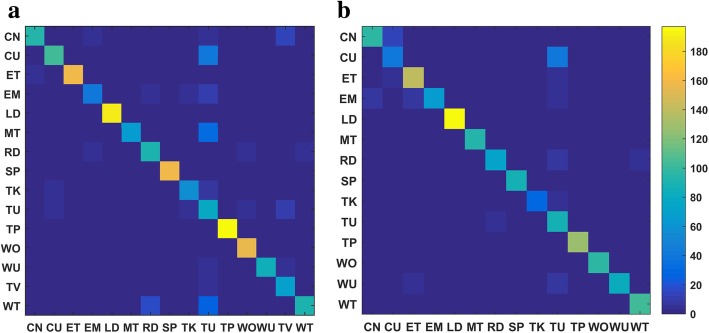
Confusion matrices for the fusion results obtained by 2-L HFNDCS as applied to **a** wearer 1 and **b** wearer 2

#### ADL recognition results using images

Bag of tags (BoTs) were extracted from all key frames in both the training and test sets using the ClarifaiNet with the “General” model [30]. In the process of obtaining the entropy-based TF-IDF classifier for the training set, the positive constant *λ* used to remap item *R*(*e*2_*i*_) was empirically selected to be 0.01 and the number of tags was *M* = 20. The confusion matrices and *F*_1_ measures of the recognition results are presented in Figs. 7 and 10, respectively.

The results in Figs. 7 and 10 indicate that the image-based method achieves fairly high recognition accuracy for ADLs with different environments and combination of objects (CoOs). In contrast, when the classifier is used to distinguish among activities with similar environments and CoOs, the recognition results are less accurate. Specifically, the following situations are notable: (1) The environments and CoOs of different activities are almost identical. For example, there is no essential difference between “reading” and “writing,” except for the presence of a pen. If this key object is not correctly recognized, it is very difficult to distinguish these two activities. (2) Although the objects in use are not the same, the BoTs extracted from these objects are very similar. For example, the BoTs extracted from “computer use” and “telephone use” are very similar, as both contain tags such as “screen,” “electronics,” and “information,” making it hard to distinguish whether the wearer is using a computer or a telephone. (3) There are overlaps among some activities. For example, overlaps occur among “meeting,” “computer use,” and “talking,” because meetings usually include operating a computer and talking, resulting in errors in some short-term recognition results. Nevertheless, there are usually differences in the duration of these competing activities; for example, computers and telephones are generally not used at the same time, and many meetings have a relatively fixed schedule. Additionally, there are some differences among the motion status of activities with similar BoTs, which can be reflected by IMU and GPS sensor data. Therefore, the accuracy of ADL recognition can be further improved by fusing the knowledge and recognition results from the sensors.

#### ADL recognition results using motion sensors

For the SVM classifier in the sensor-based method, the size of the time window for feature extraction is 3 s; the features extracted from this time window constitute a 127-dimentional vector, as described in Section 2.4. In training the classifier, the SVM uses a radial basis function as the kernel. For the training samples of W1 and W2, the cost and gamma parameters (c, g) were determined using cross-validation to be (16, 0.33) and (5.29, 0.57), respectively. The *F*_1_ measure of the sensor-based method when applied to the test datasets of the two wearers is plotted in Fig. 10.

As mentioned above, motion sensors usually offer better discrimination between activities with a clearly different motion status. As seen in Fig. 10, the motion sensor-based method achieves better recognition accuracy for activities such as “cleaning,” “lying down,” “transportation,” and “walking outside.” For sedentary activities such as “reading,” “telephone use,” “watching TV,” and “writing,” the discrimination is relatively poor. Therefore, the recognition results from the sensor-based method are not suitable for direct fusion with the knowledge and image-based recognition results; they can only be used as auxiliary evidence in the 2-L HFNDCS algorithm.

#### Fusion of three data sources using 2-L HFNDCS

After obtaining the BBAs of the image and motion sensor-based methods, the 2-L HFNDCS algorithm was applied to fuse this with the knowledge BBA. Analysis of the confusion matrices from the image-based method (Fig. 7) indicates that the most confusing activities are sedentary activities, and no more than three other activities are frequently confused with each individual sedentary activity. Therefore, in the implementation of 2-L HFNDCS, the number of candidate activities for the second-level fusion was set to Nc = 3. The confusion matrix of the recognition results after fusion using 2-L HFNDCS is presented in Fig. 8. The *F*_1_ measure of the fusion results for the three sources is illustrated in Fig. 10.

#### Fusion results of the image-based method and the sensor-based method using simplified 2-L HFNDCS

**Fig. 9:**
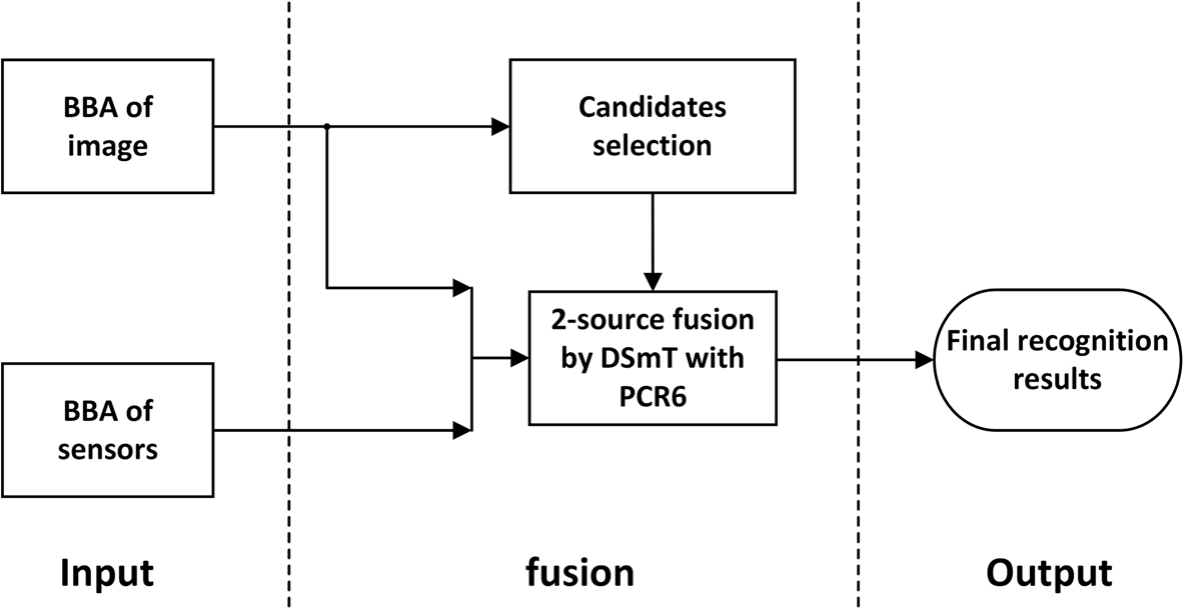
Architecture of the simplified 2-L HFNDCS

**Fig. 10:**
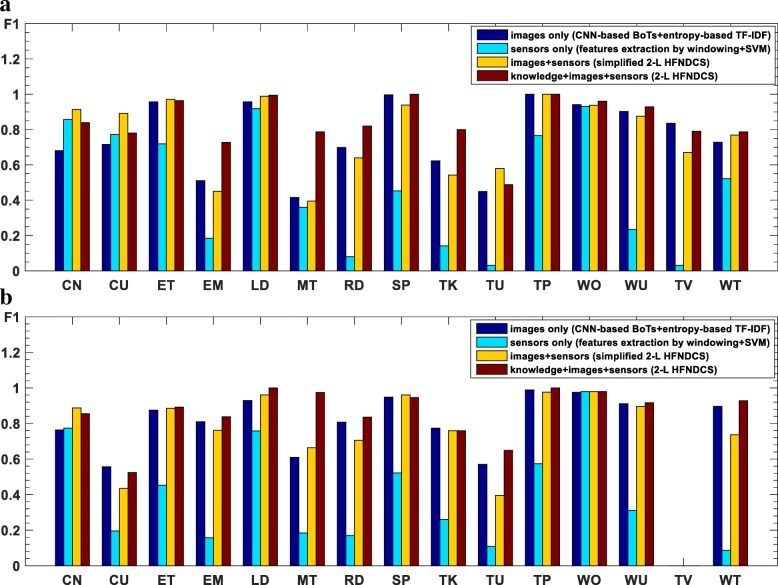
*F*_1_ measures of four methods as bar graphs for **a** wearer 1 and **b** wearer 2

To verify the effect of prior knowledge, the BBA of the knowledge data was removed so that only the image-based results and the sensor-based results were fused. The fusion process still tries to adopt the 2-L HFNDCS algorithm, but the first fusion layer is no longer needed because there is no knowledge BBA. Thus, the algorithm can be simplified. Considering the reliability difference between the image-based and sensor-based results, the process of candidate selection is retained in the second layer and candidate activities are directly selected from the image-based results (note that Nc = 3). The simplified 2-L HFNDCS without knowledge BBA is illustrated in Fig. 9. The *F*_1_ measure of the fusion results for the image-based method and the sensor-based method is also illustrated in Fig. 10. 

Comparing Figs. 7 and 8, it is clear that the recognition accuracy of confusing activities such as “entertainment,” “meeting,” “reading,” and “talking” is greatly improved when the time–activity table is added. Moreover, after fusion, the recognition accuracy for some sedentary activities that cannot be adequately distinguish by the image-based method, such as “computer use,” “telephone use,” “reading,” and “writing,” is also improved to a certain extent. In addition, as seen from Fig. 10, the image-based recognition accuracy of activities that are closely related to the motion status, such as “cleaning,” “lying down,” and “walking outside,” is also improved by the fusion with sensor-based results.

## Comparison and discussion

There are two existing ADL recognition methods that fuse egocentric visual and sensor data [15, 16]. These methods do not use a knowledge-driven model and are applicable to multimodal egocentric activity data [16] recorded by the motion sensor and video camera in Google Glass. The dataset described in [16] contains 20 different activities grouped into four top-level categories for multiple wearers (see Table 8). The method proposed in [15] performs ADL recognition by passing egocentric video through a two-stream convolutional neural network and applying motion sensor data to a multistream long and short-term memory. The recognition results are then fused by means of maximum pooling. In the method of [16], the dense trajectories of egocentric video and the temporally enhanced trajectory-like features of sensor data are extracted separately. The recognition results are then fused by a multimodal Fisher vector. As the dataset presented in [16] is openly available (http://people.sutd.edu.sg/~1000892/dataset), we can compare the results given by the proposed method with those of previous methods based on the same open datasets.

**Table 8:** Activity categories of the egocentric activity dataset presented in [15]

Ambulation	
1	Walking (WO)
2	Walking upstairs (US)
3	Walking downstairs (DS)
4	Riding elevator up (VU)
5	Riding elevator down (VD)
6	Riding escalator up (SU)
7	Riding escalator down (SD)
8	Sitting (ST)
Daily activities	
9	Eating (ET)
10	Drinking (DR)
11	Texting (TU)
12	Making phone calls (MP)
Office work	
13	Working at PC (CU)
14	Reading (RD)
15	Writing sentences (WT)
16	Organizing files (OF)
Exercise	
17	Running (RN)
18	Doing push-ups (DP)
19	Doing sit-ups (DT)
20	Cycling (CY)

### Performance comparison on their respective datasets

The proposed method was applied to the eButton datasets (described in Section 3.1), and the other two methods were applied to the dataset described in [16]. Although they were applied to different datasets, all three methods fused the vision and motion sensor data. As a result, the recognition accuracy can be compared for different information sources. The comparison results are presented in Table 9, where the average accuracy is reported over all activities and wearers.

**Table 9:** Comparison of different methods on their respective datasets

	Proposed method (%)	Method proposed in [15] (ConvNets+LSTM) + pooling fusion (%)		Method proposed in [16] (DT + temporal enhanced features) + Fisher vector (%)
		Average pooling	Maximum pooling	
Vision	79.2	68.5%	75.0	78.4
Sensors	43.1	–	49.5	69.0
Fusion	85.4	76.5	80.5	83.7

Proposed method was applied to the eButton datasets described in Section 3.1; the other two methods were applied to the datasets described in [16]

### Discussion of the comparison on the respective datasets

In Table 9, the vision-based accuracy of all three methods is similar. However, there are greater differences in the sensor-based accuracy of the proposed and existing methods, because the eButton dataset contains more sedentary activities that are difficult to distinguish using motion sensors alone, such as “entertainment,” “meeting,” and “watching TV.” Nevertheless, the accuracy of the proposed method using the fused data is higher than that of the two existing methods, mainly because our framework introduces user knowledge into the recognition process.

### Performance comparison on the same dataset

As the methods proposed in [15, 16] use egocentric video, the vision data are taken from the egocentric video in the open multimode dataset. However, the vision-based method proposed in this paper uses an egocentric image sequence, so it cannot use this open dataset directly. To enable the proposed method to be applied to the dataset in [16], we must convert the egocentric video to an egocentric image sequence. Each video and its corresponding motion sensor data are 15-s long, and the sampling rate of the motion sensor is 10 Hz. Thus, we can use the same sampling rate to convert the video to an image sequence and form a one-to-one correspondence between the images and the motion sensor data. After conversion, the egocentric image set has 20 (activities) × 10 (videos/activity) × (150 frames/video) = 30,000 frames. After extracting 20% of the key frames (6000 frames) using the key frame extraction method, two non-overlapping datasets (training set and test set) were generated (see Table 10). We define this converted dataset as $\mathcal{M}$-20.

**Table 10:** Composition of the converted dataset $\mathcal{M}$-20

Set	CY	DP	DT	DR	ET*	MP	OF	*RD*	VD	VU	SD	SU	RN	ST	*TU*	DS	US	*WO*	*CU*	*WT*
Training	150	150	150	150	*150*	150	150	*150*	150	150	150	150	150	150	*150*	150	150	*150*	*150*	*150*
Test	150	150	150	150	*150*	150	150	*150*	150	150	150	150	150	150	*150*	150	150	*150*	*150*	*150*

*Italics indicate that the activity is the same as the corresponding activity in the eButton dataset used in this paper. The same six activities constitute ${\mathcal{M}}_S$-6

Note that the methods proposed in [15, 16] do not use a prior knowledge model, and so their data (including the converted dataset $\mathcal{M}$-20) do not contain any prior knowledge, i.e., there is no corresponding time–activity table. Therefore, in applying the proposed method to $\mathcal{M}$-20, only the image and motion sensor data were fused. In addition, considering that the activities to be recognized in $\mathcal{M}$-20 are quite different from those in the eButton dataset, the same six activities were extracted from the two datasets to evaluate the ability of the proposed method to recognize the same activities in different datasets. The six activities were “eating,” “reading,” “texting” (“telephone use” in eButton dataset), “walking” (“walking outside” in eButton dataset), “working at PC” (“computer use” in eButton dataset), and “writing sentences” (“writing” in eButton dataset). The data from these activities formed a separate subset, defined as ${\mathcal{M}}_S$-6. Both $\mathcal{M}$-20 and ${\mathcal{M}}_S$-6 were used to evaluate the proposed method.

**Fig. 11:**
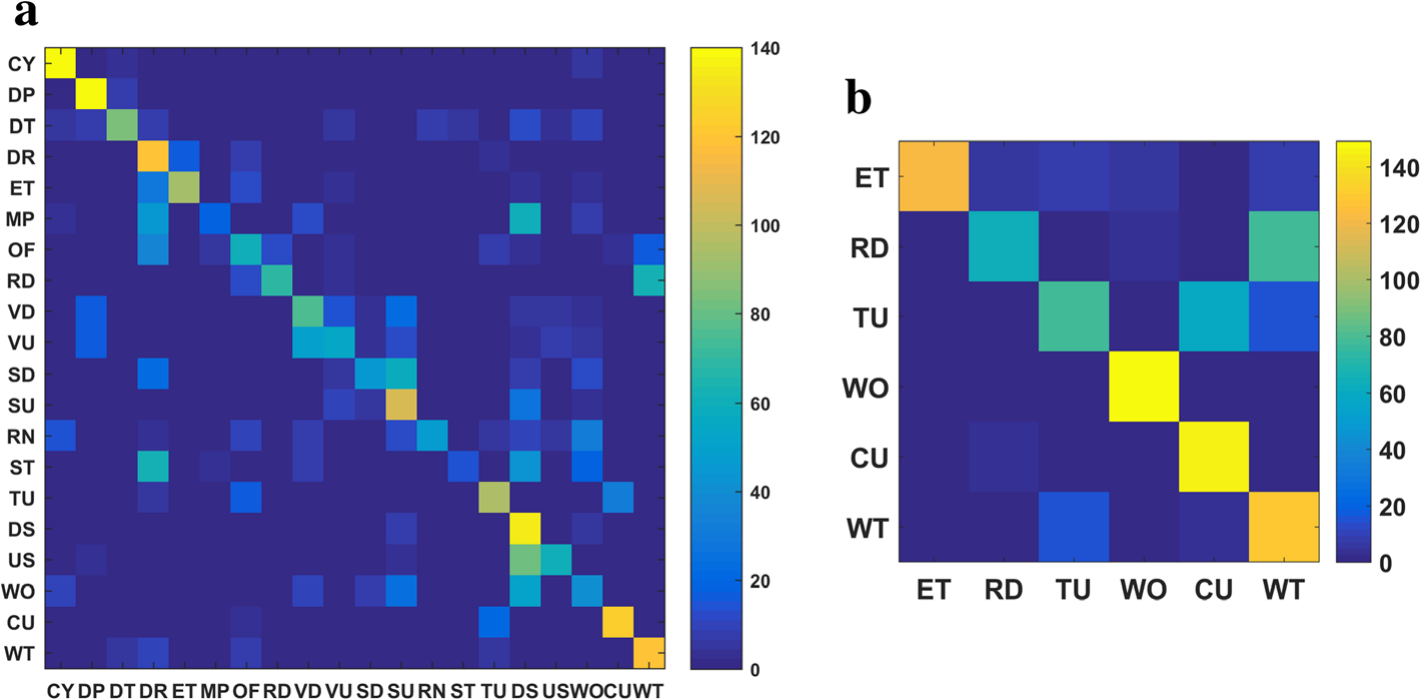
The confusion matrices of the trained BoT classifier applied to the test set of **a** $\mathcal{M}$-20 and **b** ${\mathcal{M}}_S$-6. $\mathcal{M}$-20 and ${\mathcal{M}}_S$-6 are the converted datasets extracted from the multimodal egocentric activity dataset proposed in [26]

**Fig. 12:**
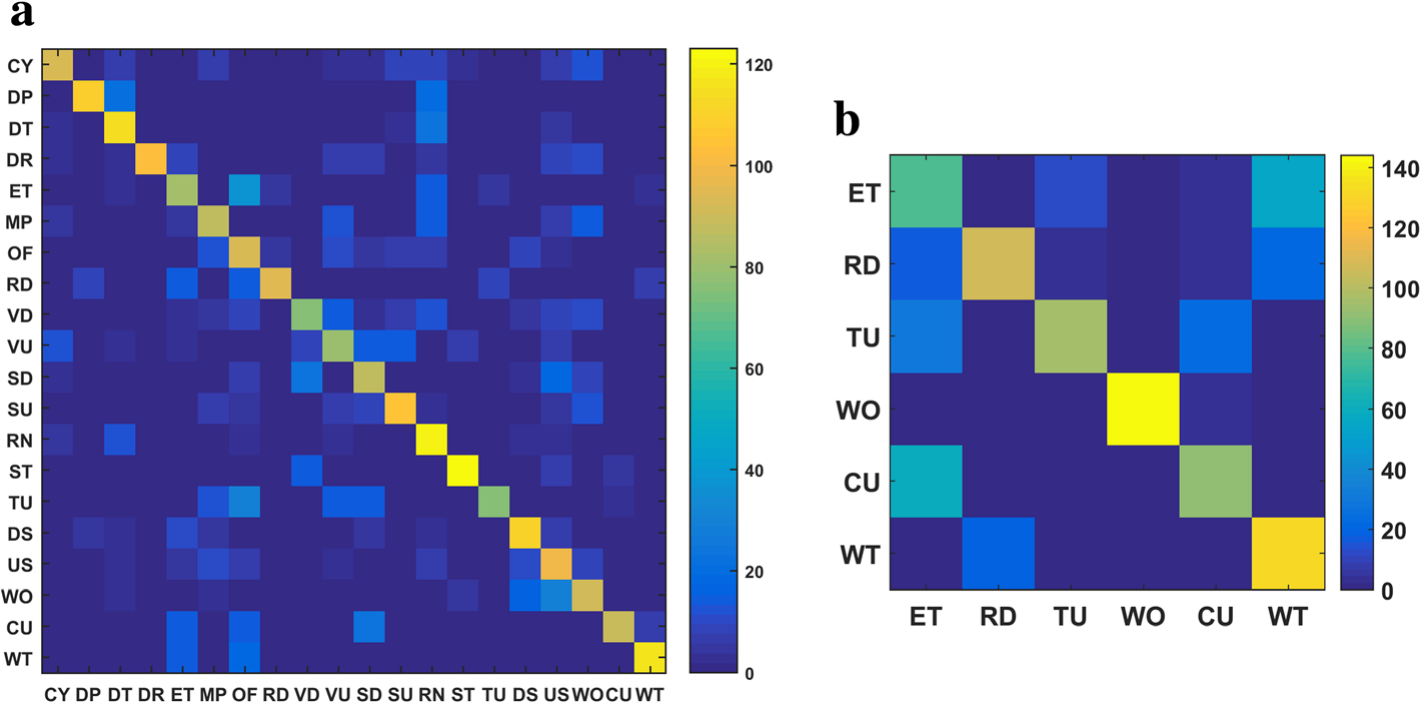
The confusion matrices of the trained SVM classifier applied to the test set of **a** $\mathcal{M}$-20 and **b** ${\mathcal{M}}_S$-6

In applying the proposed method to$\mathcal{M}$-20 and ${\mathcal{M}}_S$-6, the parameter values of the entropy-based TF-IDF algorithm used for the egocentric images in the training set are consistent with those used to analyze the eButton dataset. The confusion matrices produced by applying the trained BoT classifier to the $\mathcal{M}$-20 and ${\mathcal{M}}_S$-6 test sets are shown in Fig. 11. For the motion sensor data, feature extraction by windowing is not required because there is a one-to-one correspondence with the images produced during the conversion from video to image sequence, and the motion sensor data frame (a 19-dimensional vector) can be directly used as a feature in training the SVM. The kernel function is again the radial basis function. Using cross-validation, the cost and gamma parameters (c, g) of $\mathcal{M}$-20 and ${\mathcal{M}}_S$-6 were found to be (256, 9.19) and (5.278, 1.74), respectively. The confusion matrices produced by applying the trained SVM classifier to the $\mathcal{M}$-20 and ${\mathcal{M}}_S$-6 test sets are shown in Fig. 12.

**Fig. 13:**
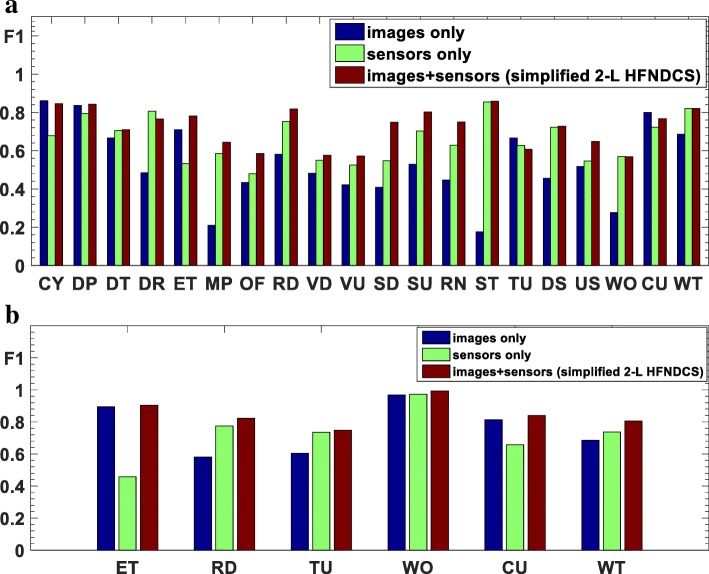
*F*_1_ measures of the fusion results for $\mathcal{M}$-20 (**a**) and ${\mathcal{M}}_S$-6 (**b**) by simplified 2-L

According to Fig. 11, the number of candidate activities in the next fusion process is Nc = 8 ($\mathcal{M}$-20) and Nc = 4 (${\mathcal{M}}_S$-6). As there is no time–activity table in the dataset, i.e., there is no knowledge BBA, the simplified 2-L HFNDCS algorithm without prior knowledge (see Section 3.2.4) was employed to obtain the fusion result of the image-based method and sensor-based method. The fused *F*_1_ measures of $\mathcal{M}$-20 and ${\mathcal{M}}_S$-6 are shown in Fig. 13. The average of the *F*_1_ measure over all activities was computed, and a comparison of the proposed method and the methods in [15, 16] on the same dataset is presented in Table 11.

**Table 11:** Comparison of different methods applied to the same dataset

	Proposed method		Method in [15]	Method in [16] (%)
	$\mathcal{M}$-20 (Nc = 8) (%)	${\mathcal{M}}_S$-6 (Nc = 4) (%)	Maximum pooling (%)	
Vision	53.2	75.8	75.0	78.4
Sensors	65.8	72.2	49.5	69.0
Fusion	72.3	85.2	80.5	83.7

### Discussion of the comparison on the same dataset

From the results in Table 11, it is apparent that applying the proposed method to $\mathcal{M}$-20 produced much lower recognition performance in the proposed vision-based method than in the existing methods. This is because the vision-based part of the proposed method is based on the combination of objects in the static images, whereas the methods in [15, 16] use vision-based motion features extracted from the video (optical flow [15] and dense trajectories [16]). Thus, for activity pairs with similar scenes but different vision-based motion features, such as “riding elevator up” and “riding elevator down,” “riding escalator up” and “riding escalator down,” “walking upstairs” and “walking downstairs,” “walking,” and “running,” the methods in [15, 16] will achieve better recognition performance. Note that, for some outdoor activities with similar scenes but large differences in motion speed, such as “making phone calls” (walking slowly while making phone calls outside), “walking,” and “running,” the proposed method can distinguish them using the speed value obtained from the GPS sensor. However, the dataset used in this experiment contains no GPS data, leading to poor recognition performance of these activities by the proposed method.

The comparison shows that the proposed method is better suited for recognition of ADLs with larger scenes or object differences. This result is validated by the high recognition accuracy of the proposed method when applied to dataset ${\mathcal{M}}_S$-6. Compared to the existing methods, the key factor in the proposed framework is the introduction of the prior knowledge model. Considering that neither $\mathcal{M}$-20 nor ${\mathcal{M}}_S$-6 contains any prior knowledge data, the recognition performance could be expected to improve significantly once the wearers’ daily routines (time–activity tables) are introduced.

In addition, for the methods proposed in [15, 16], it is necessary to extract optical flow between adjacent frames by means of the time-consuming optical flow field estimation algorithm. Even in the multistream deep learning framework proposed by [15], in the video processing part, three convolutional networks are used to accomplish feature extraction of single-frame images, optical flow, and stabilized optical flow. In contrast, the proposed algorithm only deals with a single-frame image and does not need to extract the optical flow. Only a pre-trained convolutional neural network is needed to extract the semantic features of a single image. Therefore, the computational complexity of the proposed algorithm is much lower than that of the methods proposed in [15, 16]. Detailed complexity comparison can be seen in Table 12.

**Table 12:** Comparison of the complexity of different methods

	Proposed method	Method in [15]	Method in [16]
Vision	One pre-trained CNN (single image) + entropy-based TF-IDF	Three-stream CNN (single frame, optical flow, and stabilized optical flow)	Optical flow-based dense trajectory
	Low	High	Very high
Sensors	SVM	Four-stream LSTM	Temporal enhanced trajectory-like features
	Low	Medium	Low
Fusion	DSmT	Average or maximum pooling	Multimodal Fisher vector
	Medium	Low	Medium

## Conclusion

A knowledge-driven multisource fusion framework for egocentric activity of daily living (ADL) recognition is presented in this paper. The framework is based on Dezert–Smarandache theory (DSmT) and consists of information from three sources: a set of knowledge obtained from the wearer, a set of images from a wearable camera, and a set of sensor data from an IMU and a GPS sensor. With regard to user knowledge, we propose a convenient model building method, which only requires the user to fill in a time–activity table through a user-friendly interface. For the egocentric image sequence, we propose a novel egocentric ADL recognition algorithm based on image semantic features. An advanced automatic annotation algorithm is used based on a pre-trained CNN to obtain semantic information from each image, and an entropy-based algorithm is subsequently applied to further extract semantic features, so as to reduce the image classification problem to a text classification problem. In addition, in the DSmT-based multisource fusion part, we propose a hierarchical fusion architecture to eliminate the reliability differences between different information sources. Our experimental results show that the recognition performance for a number of ADLs that have previously been considered difficult can be significantly improved through the fusion of user knowledge with information from images and motion sensors. When applied to a self-built egocentric activity dataset, the proposed method achieved an average recognition accuracy of 85.4% across 15 predefined ADL classes, significantly higher than the accuracy that can be reached without incorporating user knowledge.

## Appendix 1

### Detailed time–activity tables for wearer 1

**Table 13:** Time–activity table for workdays (Monday to Thursday) for eButton wearer 1

Time period	CN	CU	ET	EM	LD	MT	RD	SP	TK	TU	TP	WO	WU	TV	WT
0:01–6:50	0	0	0	0	10	0	0	0	0	0	0	0	0	0	0
6:51–7:20	2	2	10	0	0	0	3	0	6	8	0	0	8	0	1
7:21–7:30	0	0	0	0	0	0	0	0	6	8	0	10	0	0	0
7:31–8:00	0	0	0	0	0	0	0	0	10	8	0	9	0	0	0
8:01–8:50	1	10	0	0	0	0	0	0	5	8	3	3	5	0	0
8:51–9:20	0	5	0	0	0	0	2	0	0	9	2	10	0	0	2
9:21–10:00	0	10	1	0	0	0	5	0	6	8	0	5	3	0	5
10:01–11:20	0	10	1	0	0	0	5	0	5	8	0	0	3	0	5
11:21–12:00	0	5	4	0	0	0	2	2	3	8	0	10	0	0	2
12:01–12:30	0	2	10	0	0	0	2	0	5	8	0	4	2	8	1
12:31–13:00	0	2	2	0	8	0	0	0	5	3	0	1	0	5	0
13:01–13:30	0	0	2	0	10	0	0	0	2	6	0	0	0	4	0
13:31–13:50	0	4	0	0	0	0	4	0	0	9	0	10	0	0	2
13:51–17:00	0	10	4	0	0	0	8	0	6	8	0	5	2	0	8
17:01–17:30	0	3	2	2	0	0	1	0	8	9	0	10	2	2	1
17:31–18:00	8	1	10	5	0	0	1	0	5	8	0	3	1	1	0
18:01–20:00	4	2	4	6	0	0	10	0	8	9	0	1	0	8	5
20:01–21:00	2	4	5	10	0	0	6	0	5	6	0	0	3	8	0
21:01–22:00	2	10	1	0	0	0	3	0	5	6	0	0	3	9	2
22:01–00:00	1	10	2	0	5	0	0	0	0	5	0	0	8	9	0

**Table 14:** Time–activity table for Friday (on this day a regular meeting present) for eButton wearer 1

Time period	CN	CU	ET	EM	LD	MT	RD	SP	TK	TU	TP	WO	WU	TV	WT
0:01–6:50	0	0	0	0	10	0	0	0	0	0	0	0	0	0	0
6:51–7:20	2	2	10	0	0	0	3	0	6	8	0	0	8	0	1
7:21–7:30	0	0	0	0	0	0	0	0	6	8	0	10	0	0	0
7:31–8:00	0	0	0	0	0	0	0	0	10	8	0	9	0	0	0
8:01–8:50	1	10	0	0	0	0	0	0	5	8	3	3	5	0	0
8:51–9:20	0	5	0	0	0	0	2	0	0	9	2	10	0	0	2
9:21–10:00	0	10	1	0	0	0	5	0	6	8	0	5	3	0	5
10:01–11:00	0	10	0	0	0	0	5	0	5	8	0	0	3	0	5
11:01–11:20	0	5	4	0	0	0	2	2	3	8	0	10	0	0	5
11:21–11:45	0	2	10	0	0	0	2	0	5	8	0	0	2	8	1
11:46–12:00	0	0	2	0	0	0	0	0	0	9	0	10	0	0	0
12:01–15:00	0	2	1	0	0	10	2	0	5	8	0	2	0	0	1
15:01–17:00	0	10	4	0	0	0	8	3	6	8	5	0	2	0	8
17:01–17:30	0	3	2	2	0	0	1	2	8	9	2	10	2	2	1
17:31–18:00	8	1	10	5	0	0	1	2	5	8	2	2	1	1	0
18:01–20:00	5	2	4	6	0	0	0	6	5	5	2	0	0	4	0
20:01–21:00	2	0	0	2	0	0	0	3	5	8	2	0	1	8	0
21:01–22:00	1	0	1	1	0	0	0	3	5	6	0	0	3	9	0
22:01–00:00	3	5	2	0	2	0	0	0	0	5	0	0	8	10	0

**Table 15:** Time–activity table for Saturday for eButton wearer 1

Time period	CN	CU	ET	EM	LD	MT	RD	SP	TK	TU	TP	WO	WU	TV	WT
0:01–1:00	0	0	0	0	5	0	0	0	0	6	0	0	10	0	0
1:01–8:00	0	0	0	0	10	0	0	0	0	0	0	0	0	0	0
8:01–9:00	0	2	10	0	0	0	1	0	0	8	0	0	8	0	0
9:01–10:00	6	0	3	1	0	0	0	2	2	8	5	0	0	2	0
10:01–12:00	0	3	0	2	0	0	0	10	5	8	8	2	2	5	0
12:01–13:00	0	2	6	2	0	0	0	10	5	8	8	2	2	5	0
13:01–14:00	0	2	8	0	0	0	0	5	5	8	2	2	2	2	0
14:01–18:00	0	0	0	3	0	0	0	8	5	8	2	2	2	2	0
18:01–19:00	0	0	10	3	0	0	0	5	5	8	2	2	2	4	0
19:01–20:00	0	0	5	2	0	0	0	3	5	8	2	2	2	5	0
20:01–21:00	0	1	0	2	0	0	0	3	5	6	2	0	1	8	0
21:01–22:00	1	2	1	1	0	0	0	3	5	6	0	0	3	9	0
22:01–00:00	0	10	2	0	2	0	0	0	0	5	0	0	8	10	0

## Appendix 2

### Detailed time–activity tables for wearer 2

**Table 16:** Time–activity table for workdays (Monday, Wednesday, to Friday) for eButton wearer 2

Time period	CN	CU	ET	EM	LD	MT	RD	SP	TK	TU	TP	WO	WU	TV	WT
2:01–7:00	0	0	0	0	10	0	0	0	0	0	0	0	0	–	0
7:01–8:00	0	2	0	4	6	0	0	0	0	0	0	3	0	–	0
8:01–9:00	3	5	5	2	5	0	0	0	0	2	0	10	8	–	0
9:01–10:00	0	8	0	1	2	0	3	0	2	2	0	5	0	–	3
10:01–11:00	0	9	0	0	0	0	5	0	4	1	0	1	0	–	3
11:01–12:00	0	9	0	0	0	0	5	0	6	1	0	1	0	–	4
12:01–13:00	0	9	5	5	3	0	3	0	4	2	0	6	0	–	5
13:01–14:00	0	9	7	5	5	0	3	0	2	1	0	6	0	–	5
14:01–15:00	0	9	2	0	5	0	5	0	3	1	0	2	0	–	5
15:01–16:00	0	9	0	0	0	0	5	0	1	1	0	1	0	–	5
16:01–17:00	0	9	0	0	0	0	5	0	1	1	0	1	0	–	5
17:01–18:00	0	9	0	0	0	0	3	0	1	1	0	1	0	–	5
18:01–19:00	0	7	7	0	0	0	3	0	1	1	0	6	0	–	5
19:01–20:00	0	7	4	0	4	0	3	0	1	1	5	6	0	–	3
20:01–21:00	0	9	4	0	1	0	5	0	1	1	0	1	0	–	3
21:01–22:00	0	9	2	0	0	0	5	0	3	3	2	1	0	–	3
22:01–23:00	0	9	1	6	0	0	3	0	3	3	6	7	0	–	3
23:01–0:00	0	7	0	7	0	0	0	0	1	1	4	7	0	–	0
0:01–1:00	0	3	0	6	7	0	0	0	1	0	4	4	8	–	0
1:01–2:00	0	1	0	2	8	0	0	0	0	0	1	0	0	–	0

**Table 17:** Time–activity table for Tuesday (on this day a regular meeting present) for eButton wearer 2

Time period	CN	CU	ET	EM	LD	MT	RD	SP	TK	TU	TP	WO	WU	TV	WT
2:01–7:00	0	0	0	0	10	0	0	0	0	0	0	0	0	–	0
7:01–8:00	0	2	0	4	6	0	0	0	0	0	0	3	0	–	0
8:01–9:00	3	5	5	2	5	0	0	0	0	2	0	10	8	–	0
9:01–10:00	0	8	0	1	2	3	3	0	2	2	0	5	0	–	3
10:01–11:00	0	9	0	0	0	7	5	0	4	1	0	1	0	–	3
11:01–12:00	0	9	0	0	0	6	5	0	6	1	0	1	0	–	4
12:01–13:00	0	9	5	5	3	0	3	0	4	2	0	6	0	–	5
13:01–14:00	0	9	7	5	5	0	3	0	2	1	0	6	0	–	5
14:01–15:00	0	9	2	0	5	0	5	0	3	1	0	2	0	–	5
15:01–16:00	0	9	0	0	0	0	5	0	1	1	0	1	0	–	5
16:01–17:00	0	9	0	0	0	0	5	0	1	1	0	1	0	–	5
17:01–18:00	0	9	0	0	0	0	3	3	1	1	0	1	0	–	5
18:01–19:00	0	7	7	0	0	0	3	3	1	1	0	6	0	–	5
19:01–20:00	0	7	4	0	4	0	3	5	1	1	5	6	0	–	3
20:01–21:00	0	9	4	0	1	0	5	5	1	1	0	1	0	–	3
21:01–22:00	0	9	2	0	0	0	5	0	3	3	2	1	0	–	3
22:01–23:00	0	9	1	6	0	0	3	0	3	3	6	7	0	–	3
23:01–0:00	0	7	0	7	0	0	0	0	1	1	4	7	0	–	0
0:01–1:00	0	3	0	6	7	0	0	0	1	0	4	4	8	–	0
1:01–2:00	0	1	0	2	8	0	0	0	0	0	1	0	0	–	0

**Table 18:** Time–activity table for Saturday for eButton wearer 2

Time period	CN	CU	ET	EM	LD	MT	RD	SP	TK	TU	TP	WO	WU	TV	WT
2:01–8:00	0	0	0	0	10	0	0	0	0	0	0	0	0	–	0
8:01–9:00	0	2	0	4	6	0	0	0	0	0	0	3	0	–	0
9:01–10:00	5	5	5	2	5	0	0	0	0	2	0	10	8	–	0
10:01–11:00	0	8	0	1	2	0	3	0	2	2	0	5	0	–	3
11:01–12:00	0	9	0	0	0	0	5	0	4	1	0	1	0	–	3
12:01–13:00	0	9	5	5	3	0	3	0	4	2	0	6	0	–	5
13:01–14:00	0	9	7	5	5	0	3	0	2	1	0	6	0	–	5
14:01–15:00	0	9	2	0	5	0	5	0	3	1	0	2	0	–	5
15:01–16:00	0	9	0	0	0	0	5	0	1	1	0	1	0	–	5
16:01–17:00	0	9	0	0	0	0	5	0	1	1	0	1	0	–	5
17:01–18:00	0	9	0	0	0	0	3	0	1	1	0	1	0	–	5
18:01–19:00	0	7	7	0	0	0	3	5	1	1	0	6	0	–	5
19:01–20:00	0	7	4	0	0	0	3	3	1	1	5	6	0	–	3
20:01–21:00	0	9	4	0	0	0	5	0	1	1	0	1	0	–	3
21:01–22:00	0	9	2	0	0	0	5	0	3	3	2	1	0	–	3
22:01–23:00	0	9	1	6	0	0	3	0	3	3	6	7	0	–	3
23:01–0:00	0	7	0	7	0	0	0	0	1	1	4	7	0	–	0
0:01–1:00	0	3	0	6	7	0	0	0	1	0	4	4	8	–	0
1:01–2:00	0	1	0	2	8	0	0	0	0	0	1	0	0	–	0

## Abbreviations

2-L HFNDCS: Two-level hierarchical fusion network with descending candidate sets
ADL: Activities of daily living
BBA: Basic belief assignment
BoTs: Bags of tags
CNN: Convolutional neural network
CoO: Combination of objects
DSmT: Dezert–Smarandache theory
DST: Dempster–Shafer evidence theory
GPS: Global positioning system
IMU: Inertial measurement unit
LSTM: Long- and short-term memory
PCR: Proportional Conflict Redistribution
SVM: Support vector machine
TF-IDF: Term frequency-inverse document frequency
